# Characterization, Nutrient Intake, and Nutritional Status of Low-Income Students Attending a Brazilian University Restaurant

**DOI:** 10.3390/ijerph18010315

**Published:** 2021-01-04

**Authors:** Ygraine Hartmann, Rita de Cássia C. de A. Akutsu, Renata Puppin Zandonadi, António Raposo, Raquel B. A. Botelho

**Affiliations:** 1 Department of Nutrition, Campus Darcy Ribeiro, University of Brasília, Brasília 70910-900, Brazil; ygrainehartmann@gmail.com (Y.H.); rita.akutsu@gmail.com (R.d.C.C.d.A.A.); renatapz@yahoo.com.br (R.P.Z.); 2 CBIOS (Research Center for Biosciences and Health Technologies), Universidade Lusófona de Humanidades e Tecnologias, Campo Grande 376, 1749-024 Lisboa, Portugal

**Keywords:** body mass index, consumption, low-income students, university restaurants

## Abstract

In Brazilian universities, the university restaurant (UR) is essential in supporting students to complete their courses, as the UR offers free or low-cost food. In this sense, this research aimed to evaluate public policy effectiveness in offering food to low-income students attending the UR of the University of Brasília. This cross-sectional study compared low-income students (participating in the Student Assistance Program—Group 1) and students that did not participate in the Program (Group 2). Researchers assessed food consumption through direct observation of students while serving their plates at UR (in all meals consumed at UR) and completed food consumption with diet recalls for the meals outside the UR. In total, three complete days, including one weekend day, were evaluated for each student. Researchers also evaluated the participants’ body mass composition and body fat percentage. The results of the comparisons between the evaluated groups showed that the groups presented similar intakes. Only sodium intake was significantly different for males, being higher for Group 1. The median sodium consumption among females and males in group 1 was 55% and 119%, respectively, above the upper limit (UL). In Group 2, sodium intake levels reached consumption percentages above UL by 36% for females and 79% for males. The prevalence of inadequate sodium consumption was 100% for both genders and groups. Extra salt was added to dishes by 19.7% of the students. For females, only fiber ingestion was statistically different, with higher intake for Group 1. The other evaluated parameters showed similarities among groups for each gender. The statistical analysis revealed a significant difference in the consumption of calories, fibers, sodium, iron, and calcium for the students who had three meals at the UR in the two weekdays. There was a statistical difference in nutrients for those who had three meals in the UR, reinforcing the importance of the UR’s meals. The current food and nutrition policy at the UR proved to be extremely important in university students’ lives and in maintaining healthy nutritional aspects. However, changes in sodium use, more calcium intake, and less cholesterol consumption should receive attention to better balance dietary elements of the food offered. Dish preparation should be carefully followed to ensure the quality of the food for university students.

## 1. Introduction

Since 2010, the Brazilian government has been providing financial resources to support students from socioeconomically vulnerable groups to guarantee their permanence and graduation in Brazilian public universities. This action is established by the National Program of Student Assistance (PNAES) [1]. One of its objectives is to provide food assistance to university low-income students, given their routine on campus, long class periods and extra-class activities, the distance between their houses and the university, as well as public transportation difficulties. The government also aims to improve the health of low-income students, since food access and healthy food choices have a substantial impact on the prevention and treatment of several diseases, higher academic achievement, and lower risk of overweight [2,3,4]. Therefore, the PNAES program focuses on guaranteeing feeding’s social rights consolidated by the Universal Declaration of Human Rights [5]. 

In this sense, as part of the Student Assistance actions, the university restaurant (UR) of the University of Brasilia (UnB) serves approximately 1000 low-income students benefited daily by the National Student Program, offering free three main meals (breakfast, lunch, and dinner) from Monday to Saturday. UR meals should be nutritionally balanced, composed of fruits, raw vegetables, white and brown rice, beans, and cooked vegetables [6]. In this sense, the UR is essential for developing food policies that guarantee healthy eating and conditions of permanence and completion of the course by undergraduate students from universities [7].

Therefore, studies that analyze the quality of the offered meals in these restaurants and investigate these students’ food consumption are essential for designing intervention strategies to promote health and prevent diseases and health problems. Currently, some studies are exploring the services offered by UR in Brazil [8,9,10,11,12]. However, there is a lack of strong evidence of the nutritional status and dietary intake of low-income undergraduate students in Brazilian public universities. In this sense, this research aimed to evaluate students’ nutritional status and dietary intake participating in the Student Assistance Program from the University of Brasília (UnB—Federal District/Brazil) to look at the nutritional quality and the nutritional intake of these students’ diets.

## 2. Materials and Methods 

### 2.1. Study Design

This study was performed at the UR of the University of Brasilia, Midwest region, Brazil. In this UR, the meals are planned and supervised by the dietitians responsible for the restaurant. The Research Ethics Committee of the Faculty of Health of the University of Brasília approved this research (n. 610.774.2014). A cross-sectional epidemiological method was chosen to allocate two groups to control age variables (± two years) and gender. It increased the possibility of study participants presenting similar characteristics and the statistical tests’ power to detect differences or associations to interpret results easier. Therefore, the participants of Groups 1 and 2 were paired by gender and age.

### 2.2. Participants

Group 1 consisted of registered students who composed a low-income group that did not pay for the meals in UR (support provided by the University to guarantee student permanence and graduation). Students from Brazilian public universities are primarily from public schools and have an average income of up to 1.5 minimum wage (Brazilian minimum wage—US$289.00) to participate in this Program. Criteria for inclusion of individuals in the study were: participants had to do at least two meals on the same day at the UR (at least once, within 15 days); acknowledgment of participating in the research; to be at least 18 years old. Group 2 comprised students who did not participate in the PNAES program but had meals at the UR. Pregnant females were excluded from the study since they have different nutritional needs and body composition from the general population.

There were a total of 439 registered individuals with characteristics of Group 1 in the university system. They were contacted via email. In the electronic message, the student was invited to participate in the research and answer a brief questionnaire to outline the socioeconomic profile. Of the 439 individuals invited to participate in the survey, 33.94% (*n* = 149) answered the questionnaire. There were three attempts through email for individuals to answer the survey. After contacting them, the final sample of active participants until the end of the research (all steps completed) in Group 1 was 79. Group 2 included students from the University who did not participate in PNAES but attended the UR to have meals, lunch and/or dinner. Group 2 had a family income higher than 1.5 minimum wage (about US$435.00) since they were not part of PNAES. Students from Group 2 also needed to be at least 18 years old and agreed to participate in the research.

For Group 2, the selection criteria at the cafeteria line followed methods previously established [10,13], using a systematic order to approach users, one user for every 15 that entered the UR on the first day of data gathering. Researchers invited 149 students, the same number invited in Group 1, since participants could not complete the study’s three days. Group 2 answered the same questionnaire as Group 1 after acceptance to participate in the research on the first day of observation. Students from this group received information that they needed to come to the UR for a second and third day. They should identify themselves to researchers in the area designed for data gathering. Therefore, the final sample for this group was 94 individuals. For both groups, the final sample represented the number of students who completed the whole study protocol, questionnaire answering, weight, height, body fat measures, and three days of food intake for nutritional analysis. The minimal number of participants for both groups with a 95% confidence interval and an error of 5% was 158 students (minimum sample of 79 students for each group). The number of students was based on a similar sample considering age and income through a pilot test conducted in the UR with other students that ate at the restaurant and were offered the same type of menu. The variance of student consumption in the pilot study was considered to determine the study sample.

### 2.3. Variables

The socioeconomic and anthropometric variables were weight, height, gender, age group, body fat, income, and participation or not in UR assistance group. To analyze the food consumed in the UR, researchers entered the production area to follow all the recipes and develop the Technical Preparations Files (TPF) [14]. These files contained data from the ingredients (type and amount used), the steps to prepare the recipe, the weight of the final recipe, portion numbers, and portion size [14]. Portion size was established by researchers (before the service) for each dish with the buffet’s available silverware. The researchers classified the portions as small (S), medium (M), or large (L), registering the weight and size to use in the observational data collection. This procedure was proposed to guarantee preciseness regarding the nutrients offered in UR meals. In Brazil, lunch and dinner are the main meals and represent about 70% of the population’s daily intake [15]. After collecting these data, we inserted each TPF’s (dish) accurate information in the Dietwin^®^ software to perform nutrient analysis. The Toledo 9091^®^ scale (100 kg capacity and 20 g precision) was used to weigh the production area dishes. These procedures occurred during the study and for all the meals served at the UR. With all the calculated TPF and portions, meals performed at the UR could be nutritionally evaluated in this study.

Upon arriving at the UR, students entered the cafeteria line and served themselves (self-service). It is important to mention that meat dishes and fruits (as a dessert) are traditionally portioned and served by restaurant employees for lunch and dinner at the UR. Therefore, a trained researcher observed each student from a distance, as he/she served himself/herself at the distribution counter, writing down each of the served dishes portions (including desserts and drinks). This procedure was followed up by the researchers who classified the food portions served by the participants into a standard unit (U), small (S), medium (M), or large (L), according to the methodology proposed by Savio et al. [15]. After assembling the plate, the meal served on the plate was weighed (FILIZOLA^®^ scale, capacity of 20 kg) and noted on a specific form containing the participants’ identification, the empty plate’s weight, and the weight of the plate with the portioned food. Items served outside the dish assembly were also weighed, such as salads and soups served separately, drinks, desserts, bread, spices, olive oil, sauces, and cassava flour. The added salt to the meal was also considered. Participants received extra 1 g salt packages that were available at the end of the counter. Researchers counted the number of bags each individual got before sitting to do their meals.

After the meal, the participant returned his/her plate to be weighed again to assess leftovers. For food waste, items that composed the plate were proportionally deducted, considering the percentage of each preparation in the composition of the plate. When the menu contained chicken with bones, 17.5 g of bone per serving (average bone weight) were deducted from the dish’s weight and the waste.

All the described procedures were necessary to evaluate food intake in the UR for both groups. Students from Group 1 performed their three main meals at the UR during the week. For Group 2, students had lunch and dinner at the UR during the week. While they were eating meals at the UR, we asked the participant about food consumption performed outside the UR, through a 24 h recall (R24) instrument [16]. This procedure was important to complete the daily food intake with meals not performed at the UR during the week and get full information on the consumption during one day of the weekend. Therefore, complete R24 was also used to evaluate food consumption on the weekend day. For the two weekdays, a recall was used for meals performed outside the UR to obtain complete data on the individuals’ diet. In the end, three days of food intake were accomplished for each student. Food weighing is the gold standard for dietary intake evaluation [17], and with the recipes of the preparations from the UR, researchers had more precise data on nutrient intake. However, not all the meals were eaten at the UR, and researchers had to combine two methods to investigate the whole day of consumption and also the weekend intake.

The parameters proposed by the Institute of Medicine were used to assess the adequacy of food intake for micronutrients and cholesterol [18,19]. The nutritional variables analyzed corresponded to the ingested values of macronutrients, calcium, fibers, iron, sodium, and cholesterol, as well as the total energy intake (TEI) and TEE (total energy expenditure) [20]. The adequacy of iron and sodium intake was assessed using the estimated average requirement (EAR) and upper limit (UL) methods as cutoff points, according to sex and age group.

The anthropometric data assessments were scheduled according to each participant’s convenience and performed in a private room inside the UR. For anthropometric evaluation, weight measurements (G-tech scale, model: Glass 200^®^ with a capacity of 200 kg) and height were collected, and based on these data, the body mass index (BMI) was calculated [21]. For body fat (BF) percentage, researchers used Biodynaics^®^ BIA 310 Bioimpedance analyzer (WA, USA) and the protocols recommended by Pollock and Wilmore [22]. After the evaluation, body percentage was calculated and classified according to the cutoff points proposed by the American Dietetic Association (ADA)/Canadian Dietetic Association (CDA) (high %BF ≥ 25 for male and %BF ≥ 30 for female) [23].

Along with food intake information, participants were asked about physical activity in the last seven days. For this, the instrument The Stanford 7-Day Recall (7-DR) was used [24] to investigate the performance of moderate, intense, and very intense physical activities, in addition to sleep time, during the seven days before the interview. These data were used to achieve the total energy expenditure (TEE) of individuals [20].TEE was calculated according to Institute of Medicine (IOM) and the average hours per day in activities were considered using metabolic equivalent intensity (MET) values (sleep = 1, light = 1.5, moderate = 4, hard = 6, and very hard = 10) [24].

### 2.4. Statistical Analysis

Statistical Package for Science—SPSS Program version 20.0^®^ (IBM Corporation; New York, NY, USA) was used to analyze data. After creating the data entry form, the data was checked using frequency distribution analysis, comparing each variable’s values in the SPSS database with those of possible occurrence, avoiding typing errors. The normality assumptions were verified, and the measures of central tendency and variance of the sample were determined. Chi-square test was used to compare proportions between groups by gender. Mann–Whitney U Test was performed to verify nutrient intake considering the percentiles, since normality was not verified. For analysis, a significance level of 5% was used. The power of the study for nutrient intake was 0.987, considering sex.

## 3. Results

Table 1 shows the characterization of the participants by gender. Group 1 (*n* = 79) presented 45 males and 34 females participants with no statistical difference between them (Chi-square test; *p* = 0.216). Group 2 (*n* = 94) presented 52 males and 42 females with no difference (*p* = 0.302). Most of the participants were aged from 20 to 29 years old (median age 21, 95% CI, 18; 48), with adequate weight (61.9–69.2%) (median BMI 21.8, 95% CI, 16; 37.7). However, among females from Group 1, we found a high prevalence (26.4%) of underweight low-income, almost three times higher than females from Group 2, statistically different. Despite the lower prevalence of underweight among males, Group 1 also showed almost three times higher underweight than Group 2 males, but not significantly different. Groups 1 and 2 presented similar results (*p* > 0.05) with body fat percentage, 20.7 ± 8.9% and 19.6 ± 7.2%, respectively (Table S1). The anthropometric profile, age group, body fat percentage, and income variables of each group are presented in the Supplementary file (Table S1). Since body fat percentage differs from male and female, researchers evaluated differences between underweight/adequate weight and overweight/obese by gender.

Lunch and dinner during the weekdays happened in the UR. Therefore, researchers could compare the plates’ average weight and the percentage of leftovers for both groups. Group 1 plates’ weight was 455.6 g ± 133.9 for lunch and 413.3 g ± 162.8 for dinner with mean leftovers of 9.1%. For group 2, lunch represented 478.9 g ± 187.5 and dinner, 461.6 g ± 194.8, with mean leftovers of 7.5%. For all analyses, *p*-values in all comparisons were >0.05; therefore, there was no significant difference between the groups for meal weight and the leftovers.

The comparisons between the evaluated groups (Table 2) showed that the groups were homogeneous. Only sodium intake was significantly different for males from Groups 1 and 2, higher for Group 1. The median sodium consumption among females and males in Group 1 was 55% and 119%, respectively, above the upper limit (UL) (2300 mg). In Group 2, sodium intake levels reached consumption percentages 36% above UL for females and 79% for males. The prevalence of inadequate sodium consumption was 100% for both genders and groups. In the sample studied, 19.7% (*n* = 34) added salt (sachet 1 g) in one or more meals. For females, only TEI and fiber ingestion were statistically different, with higher intake in Group 1. The other evaluated parameters showed similarities among groups for each gender.

There was no difference among groups considering total energy intake and total energy expenditure (Table 3). Also, there was no difference between groups and underweight/adequate weight individuals and overweight/obese ones in each group (Table 3).

A comparative analysis between students who consumed the three main meals offered in the UR on Monday and Tuesday and those who did not have the three main meals is presented in Table S2. This comparison was made to obtain data that could accurately analyze those students who had their meals at the UR at all times. The statistical analysis revealed a significant difference in the consumption of calories, fibers, sodium, iron, and calcium for the students who had the three meals at the UR in the two weekdays. There was a statistical difference for all nutrients for those who had three meals in the UR, on both days.

When comparing energy intake and nutrients from the weekend and weekdays (Table 4), there was a statistical difference in sodium and carbohydrate consumption only within Group 2 (*p* = 0.000), the medians being higher during the weekdays. For Group 1, there was no statistical difference for these same nutrients (*p* = 0.978). There were no statistical differences for both groups for lipids and proteins when comparing weekends and weekdays (*p* = 0.978).

When comparing Groups 1 and 2, on the weekend, there were no statistical differences for energy intake (*p* = 0.500), carbohydrates (*p* = 0.640), protein (*p* = 0.665), lipids (*p* = 0.727), and sodium (*p* = 0.392). However, for the weekdays, the groups presented different intakes for energy (*p* = 0.001), carbohydrates (*p* = 0.000), protein (*p* = 0.000), and sodium (*p* = 0.000). Group 1 showed higher consumption for these mentioned nutrients and energy intake.

## 4. Discussion

Food and nutrition are essential requirements for promoting and protecting the health and enhancing human growth and development with quality of life and citizenship. Healthy eating, understood as a human right, comprises a dietary pattern appropriate to individuals’ biological and social needs according to life stages. Additionally, healthy eating should be based on eating practices assuming the sociocultural meanings of food as a basic conceptual foundation [25]. Healthy eating depends on the types of food, the form of preparation, the time to prepare it, and the time to eat. In this perspective, the university student does not always have family support for the acquisition and preparation of food. In Brazil, university students’ eating habits are strongly influenced by factors such leaving their parents’ homes due to the location of the institution, the lack of time to prepare full meals, influencing food choice, the replacement of meals with quick and practical and unhealthy snacks, and establishment of new behaviors and social relationships [26]. A narrative literature review [27] analyzed studies on university students’ food consumption, evaluating 37 articles (eight conducted in Brazil). The study showed that most university students have unhealthy eating behaviors, such as high consumption of fast food, snacks, sweets, and soft drinks, and low consumption of fruits, vegetables, fish, whole grains, and legumes. Food consumption of university students was characterized as unhealthy, regardless of undergraduate course and sex, especially those students who left their parents’ homes and became responsible for their own food [27]. The studies conducted in Brazil evaluated food consumption using food groups or food frequency questionnaires. None of the studies evaluated the meals served inside the UR through TPF, or used 24 h recalls like our study.

Since most public universities in Brazil present UR, the evaluation of the served meals is crucial to ensure that these restaurants contribute to better eating habits inside the campus. URs inside the public universities are part of the National Student Assistance Program to support low-income students. However, they should also provide nutritionally balanced meals to all the students belonging to the universities. They are spaces to guarantee the offer of fruits, vegetables, and whole grains as healthy options.

Other studies show that university spaces are important scenarios for nutritional and educational strategies because students spend much time in their days inside the campus [28,29,30]. Unlike the public policies created in Brazil for younger students, the country lacks specific public policies for university students regarding healthy eating and interventions.

According to Pereira-Santos [31], university entrance poses new responsibilities to students regarding food, housing, and financial management. It brings risk factors of an inappropriate lifestyle (sedentary lifestyle, psychosocial factors, and high demand of academia) that can contribute to the adoption of nutritionally unbalanced eating habits. Therefore, the risk of developing chronic, noncommunicable diseases increases, and the Brazilian UR plays a fundamental role in the access and the quality of the students’ meals, mainly for the low-income ones.

Our study evaluated the characterization, nutrient intake, and nutritional status of low-income students attending a Brazilian UR. Identifying it is an important strategy for promoting balanced nutrition during graduation [31]. Income can influence dietary intake, and among university students, poor diets can be associated with poorer health, lower academic achievement, and increased risk of being overweight. Our data allowed us to show the commitment of UR from the University of Brasilia to improve dietary intake to low-income students and guarantee the human right to food access [5]. It is worth to mention that all the public universities in Brazil have the same Assistance program for low-income students. Therefore, our results can represent this group of students in the country. The Education Ministry of Brazil defines the PNAES program. The criteria are the same for all the national universities, a condition being to receive meals based on the family’s low income [1].

Differently from findings shown in a recent systematic review, in which university food environments are potentially contributing to inadequate dietary intakes, and overweight/obesity risk [32], our study showed that most of the low-income students with adequate weight present adequate dietary intake regarding TEI, macronutrients, and iron. However, they presented inadequate intake regarding sodium, calcium, and cholesterol. A study conducted by Petribú et al. [33] in a sample of 250 Brazilian university students from Recife/PE showed high cholesterol consumption (>300 mg/day) among about 40% of participants, higher than the values found in our study (Table 2). It is essential to highlight that in Group 1, the TEI and TEE were similar, showing a balance of consumption by the low-income who attended the UR. Also, there was no difference between underweight/adequate weight individuals and overweight/obese ones in each group (Table 3), probably due to the similarity in body fat percentage between BMI groups for males (*p* = 0.325) and females (*p* = 0.946).

Considering BMI, the prevalence of adequate weight classification for females (Table 1) follows the data presented by the Brazilian Family Budget Research [34] (61.4%) at a similar age. Among males, our findings (68.9 and 69.2%) for adequate weight were higher than the Brazilian population [34] and higher than the study of Petribú et al. in a sample of Brazilian university students from Recife/PE [33]. In Group 2, among males, there was a prevalence of 27% of individuals with excess weight. In this group, the overweight prevalence was close to the Brazilian population (27.3% at a similar age) [34]. Similarly, females in the same group showed 28.6% overweight/obesity. Our study showed males with BMI higher than females, as well as in a study conducted by Petribú et al. (mean BMI as 20.9 kg/m^2^ for females and 23.9 kg/m^2^ for males) [33]. The proportion of excess weight found in females (5.3%) was significantly lower (*p* < 0.01) than in males (35.5%) [33]. Our data from Group 1 were similar (females with a lower proportion of excess weight than males). However, in Group 2, it was not observed. It is important to highlight that the Petribú et al. [33] study did not evaluate family income.

It is noteworthy that the percentage of underweight individuals found among females (26.4%) and males (11.1.%) in Group 1 was higher than the data found in the Brazilian population (1.8% for men and 3.5% for females) [34]. The low-income participants in our study could explain this data. However, our data revealed that they presented adequate nutrient intake, supported by UR. Underweight individuals among females were more frequent than among males for both groups, since thin body image may more significantly influence eating behavior among females than males [35]. However, it is important to highlight that this comparison was performed using only BMI. This population, being young, tends to present a high percentage of muscle weight, confirmed by the evaluation of body fat percentage. Statistical differences for males comparing BMI groups (*p* = 0.325) and females (*p* = 0.946) were not found.

Assessment of total energy intake (TEI) and total energy expenditure (TEE) is important to understand the energy balance and associate weight and health problems [35]. Males had higher median energy consumption than females for both groups (Table 2), as expected, since their TEE was also more elevated. Although there were more underweight Group 1 university students, they showed similar energy consumption to Group 2 for males, but not for females (*p* < 0.05). Brazilian females and males of similar age had an average caloric intake of 1688 kcal and 2112 kcal, respectively [34]. Therefore, the median TEI consumption for Group 2 females was similar, and for the other groups, the median of TEI was higher (Table 2) than the general Brazilian population of similar age [34].

The macronutrient distribution for both groups followed the parameters established by the IOM [20], which was a positive factor in assessing the quality of the caloric distribution for the students. The consumption of fiber in both groups (Table 2 and Table 3) was higher in the Midwest Brazilian region (in which the present study was performed), among males it averaged 23.3 g and among females 18.4 g per day [34]. However, in our study, females presented lower fiber ingestion than males (Table 2). Beans, a fiber source, may contribute to the high consumption of fiber in the population studied, since the combination of rice and beans is typical of the Brazilian diet and present daily at the UR [36,37,38]. According to our data, only three students did not consume rice, and one did not eat beans on any day of the survey. Hartmann et al. [10], in a study in the same UR, demonstrated that students ate more fruits and vegetables in meals at the UR. Authors showed that 51.7% of male and 35.8% of female low-income students consumed more than 400 g of fruits and vegetables in one day, also contributing to high amounts of fiber in the diet. Therefore, menu planning, including fruits, vegetables, and beans, is an important strategy to encourage consumption, and it is a way of nutritional education inside the UR.

The average consumption of calcium in the Midwest region [34] at a similar age was 516.6 mg/ day for males and 459.5 mg/day for females. Table 2 presents the median calcium consumption data above those found for the Brazilian population. However, our data showed that neither gender nor group reached the reference value (EAR) of 800 mg/day [18]. In both groups, females had a prevalence of inadequate calcium intake of 20.5% and 24.2%, respectively. For students who attended the UR (Group 1), this consumption deficiency can be solved with nutritional education actions that encourage milk and dairy product consumption for breakfast. In Table S2, when calcium is assessed in participants that eat the three main meals in the UR, students who consumed all three meals in the UR had calcium intake above the EAR, higher than the group that did not eat three meals at UR. Breakfast at the UR made a difference for this nutrient.

The average consumption of iron in both groups reached the nutritional recommendation [18] (Table 2). In both groups, average daily sodium consumption exceeded those found by the Brazilian Family Budget Research [34]. The high sodium consumption rate was also found in the low-income Brazilian population consumers of community restaurants [36]. Since only 19.54% (*n =* 174) of the studied individuals used added salt, it cannot be concluded that the excess sodium consumption was only due to this practice, but probably due to the lack of control over the use of ingredients rich in sodium in meal preparation. When students who had three meals in the UR are stratified (Table S2), it is possible to see an intake of approximately 1000 mg more sodium for those who had three meals in the UR. Moreover, during the week, sodium intake in Group 1 was significantly higher than during the weekend and higher than sodium intake in Group 2. This data reinforces the need to evaluate what is being served inside the UR by following the preparation techniques and ingredients [39]. Furthermore, as our study pointed out, greater use of salt and/or products rich in sodium interventions are necessary to train UR workers to reduce sodium adding.

This result is worrying. It requires intervention in the restaurants’ culinary practices to ensure compliance with the WHO recommendation [40], since restaurant users are exposed to high sodium levels in the food offered.

The benefits of reducing salt are clear and consistent and should be encouraged to prevent circulatory diseases [41]. Thus, immediate intervention is necessary, as UR diners are being subjected to excessive mineral levels, despite ready seasonings or salt-based seasonings not being used. A Brazilian study also revealed excess sodium in university students’ diets, with 83% of the studied population consuming amounts above the recommendation [8].

Cholesterol intake in the Brazilian Midwest region population presented an average of 273.5 mg for males and 206 mg for females [34]. Our data showed that cholesterol consumption was higher than the regional average. The high intake of cholesterol was also a finding in other studies among university students, which the authors attributed to excessive consumption of fast food or snacks [33,42]. Our data from 24 h recall suggested that the consumption of cholesterol may be partially associated with the consumption of beef or pork offered in the UR dishes. The choice of the type of meat and the portions needs to be carefully evaluated for possible menu changes.

The statistical analysis did not identify significant differences in the pattern of food consumption in the analyzed variables. This result was expected, considering that the two groups had a similar nutritional status, approximately the same age, and routine activities that involved similar tasks. Food in the UR did not differentiate consumption or students’ nutritional status in those who participated in the research. This result can be attributed to the large supply of fruits and vegetables on the menu, as our previous study has shown [10].

Our study presents some limitations, such as the use of 24 h recall, since its success depends on the participant’s memory, cooperation, and communication ability. However, since we used trained interviewers, this bias has been minimized. Our study’s potential strength is that although we could have performed the whole study using just the 24 h recall, we were inside the UR to get the participants. It was an opportunity to evaluate the service by creating the dishes’ Technical Preparation Files using their recipes and weighting the participants’ plates. Therefore, we had the gold standard to assess the main meals (lunch and dinner—about 70% of the participants’ intake) during the week. Since we could not weigh foods from the other meals (snacks and breakfast—about 30% of daily intake), we completed the daily intake with the 24 h recall. Also, the cross-sectional design presents its limitation, since data is obtained at a single moment in time. However, it allows characterizing the population and identifying associated factors, essential in elaborating UR strategies for the students’ access to healthy food. Also, using BMI as a tool for overweight could be a potential limitation when studying a young population, due to students with high muscle weight that can be mistaken for overweight. It was confirmed when we evaluated differences between underweight/adequate weight and overweight/obese by gender, showing no statistical differences for males comparing BMI groups (*p* = 0.325) and females (*p* = 0.946).

## 5. Conclusions

The provision of meals at UR fulfills its role of ensuring adequate energy and macronutrient intake for low-income students subjected to the restaurants’ meals. Other than the balanced offer of macronutrients, UR provides free consumption of vegetables and offers fruits as dessert. Compliance with the recommendation of the analyzed micronutrients was achieved, except for calcium and sodium. However, students that had breakfast at the UR reached higher calcium intake, showing the importance of this meal for low-income students. Nutritional education actions are essential for students and food handlers at the UR, and recipes can be modified for sodium content. Sodium majorly came from the addition of salt to preparations, not the use of industrialized products rich in sodium. Therefore, training is fundamental to improve menus, and students need to train their palates to accept lower sodium amounts in their plates. Sodium adequacy needs time, because it can not be drastically reduced without food rejection. The current food and nutrition policy at the UR proved to be extremely important in university students’ lives, ensuring a balance between TEI and TEE. The UR must have strict guidelines for healthy eating, considering that students who use it can develop better or worse health conditions due to the meal offered and consumed. Thus, the correction of nutrient indices such as sodium, calcium, and cholesterol should receive attention to better balance the nutritional aspects of the food offered. Dietitians at the UR need to pay more attention to preparing meals, guiding food handlers, and helping students select their meals.

## Figures and Tables

**Table 1:** Age group and body mass index of the participants from the University of Brasilia by gender.

Gender											
		Male					Female				
		Group 1 (*n =* 45)		Group 2 (*n =* 52)			Group 1 (*n =* 34)		Group 2 (*n =* 42)		
		*N*	%	*N*	%	*p*-Value	*N*	%	*N*	%	*p*-Value
Age group	<20	12	26.7%	12	23.1%	1.000	9	26.5%	12	28.6%	0.513
	20 to 29	30	66.7%	35	67.3%	0.535	24	70.6%	28	66.7%	0.579
	30 to 39	3	6.7%	5	9.6%	0.480	0	0.0%	0	0.0%	----
	>40	0	0.0%	0	0.0%	---	1	2.9%	2	4.8%	0.564
BMI *	Underweight	5	11.1%	2	3.8%	0.102	9	26.4%	4	9.5%	0.035 *
	Adequate weight	31	68.9%	36	69.2%	0.216	22	64.7%	26	61.9%	0.170
	Overweight	6	13.3%	11	21.2%	0.782	2	6.0%	10	23.8%	0.257
	Obesity	3	6.7%	3	5.8%	0.317	1	2.9%	2	4.8%	1.000

Chi-square test, * *p* < 0.05. Body mass index (BMI) followed the criteria adopted by the World Health Organization (WHO) [21] underweight (BMI < 18.5 kg/m^2^), adequate (BMI between 18.5 and 24.9 kg/m^2^), overweight (BMI between 25 and 29.9 kg/m^2^), and obesity (BMI ≥ 30 kg/m^2^).

**Table 2:** Central tendency and variance of nutritional composition of intake by sex and group of university students from university restaurant (UR).

	Males			Females		
	Group 1 Median (Percentile 25–75)	Group 2 Median (Percentile 25–75)	*p*	Group 1 Median (Percentile 25–75)	Group 2 Median (Percentile 25–75)	*p*
TEI (kcal/day)	2636 (2237–3173.9)	2598.0 (1833.5–3384.9)	0.405	1907.4 (1681.0–2200.9)	1644.9 (1321.2–2059.42)	0.041 *
TEE (kcal/day)	2624 (2279–2877.0)	2744.3 (2267.7–3121.4)	0.653	1850.2 (1616.3–2178.8)	1945.1 (1815.3–2052.5)	0.434
Cholesterol (mg/day)	226.4 (169.4–329.3)	234.5 (136.2–312.4)	0.862	189.3 (145.2–202.8)	159.4 (122.4–249.0)	0.352
Fiber (g/day)	51.7 (40.9–67.2)	52.0 (33.9–71.7)	0.500	35.8 (24.8–44.4)	25.0 (18.3–39.2)	0.040 *
Iron (mg/day)	17.9 (14–24.4)	16.6 (11.3–23.0)	0.275	12.9 (10.0–15.6)	10.8 (6.9–15.2)	0.053
Sodium (mg/day)	5030.8 (4470.5–6427.1)	4113.1 (3080.5–5706.7)	0.003 *	3561.6 (2925.1–3900.1)	3117.7 (2451.0–3823.5)	0.085
Calcium (mg/day)	793.7 (613.5–1134.2)	693.7 (497.1–1028.4	0.195	596.1 (476.4–726.3)	445.7 (338.5–720.1)	0.064
Carbohydrate (% of TEI)	57.8 (52.7–61.1)	57.9 (53.7–63.5)	0.365	58.1 (53.0–63.8)	56.2 (52.6–60.8)	0.444
Lipid (% of TEI)	25.0 (21.5–28. 9)	24.6 (20.2–27.6)	0.383	24.0 (20.8–27.5)	26.2 (22.6–28.0)	0.241
Protein (% of TEI)	17.3 (15.8–18.3)	16.2 (13.4–18.3)	0.2	17.1 (14.8–19.5)	16.7 (13. 8–18.9)	0.591

* *p* < 0.05; Mann–Whitney U test; TEI: total energy intake; TEE: Total energy expenditure.

**Table 3:** Total energy intake and total energy expenditure comparison between groups and underweight/adequate weight and overweight/obese individuals.

		Group 1		Group 2	
		Median (CL Inferior 95.0% CL Superior 95.0%)	*p*-Value	Median (CL Inferior 95.0% CL Superior 95.0%)	*p*-Value
**TEI (Kcal)**	Underweight/adequate	2707.2 (2443.3–2963.2)	0.564	3153.6 (2543.0–3649.5)	0.526
	Overweight/obesity	2558.8 (1930.9–3849.1)	0.896	2245.7 (1955.8–2992.1)	
**TEE (Kcal)**	Underweight/adequate	2308.4 (2073.5–2475.6)	0.363	2476.7 (2052.5–2788.9)	0.969
	Overweight/obesity	2219.1 (1903.2–2925.6)	0.786	2182.2 (1904.2–3140.9)	

Mann–Whitney U test.

**Table 4:** Comparison of energy intake and nutrients from the weekend and weekdays.

	Group 1	Group 2
	Median (Percentiles 25–75)	Median (Percentiles 25–75)
TEI (kcal/day)-weekend	2085.7 (1561.3–2714.5)	2283.9 (1473.1–2852.6)
TEI (kcal/day)-weekdays	2339.4 (1950.5–2772.5)	1914.0 (1428.9–2617.7)
Carbohydrates (g) weekend	71.6 (49.4–106.2)	67.3 (51.8–99.0)
Lipid (g) weekend	15.9 (10.2–25.7)	15.6 (8.9–26.1)
Protein (g) weekend	22.7 (13.9–32.6)	22.0 (15.6–33.1)
Carbohydrates (g) weekday	103.8 (80.1–122.8)	84.6 (45.3–111.8)
Lipid (g) weekday	18.3 (15.1–23.1)	22.1 (13.5–51.6)
Protein (g) weekday	33.9 (27.1–39.4)	23.1 (14.1–33.8)
Sodium (mg) weekend	3312.1 (2458.7–5044.2)	3166.6 (2015.1–4988.5)
Sodium (mg) weekdays	4844.9 (3451.3–6031.3)	3598.4 (1980.5–4848.4)

## Data Availability

Suggested Data Availability Statements can be found at https://www.mdpi.com/journal/ijerph/instructions#suppmaterials.

## Supplementary Materials

The following are available online at https://www.mdpi.com/1660-4601/18/1/315/s1. Table S1. Anthropometric profile, age group, body fat, and income variables for both groups of students, consumers at the university restaurants.; Table S2. The measure of central tendency and variance of the student’s intake nutritional composition by frequency of consumption in the university restaurant.
