# Supplementary material for: Characterization, Nutrient Intake, and Nutritional Status of Low-Income Students Attending a Brazilian University Restaurant
Source: Int J Environ Res Public Health. 2021 Jan 4;18(1):315. doi: 10.3390/ijerph18010315 (PMC7795120; doi:10.3390/ijerph18010315)
Supplement: Supplementary file 1 [file ijerph-18-00315-s001.pdf]

Table S1. Anthropometric profile, age group, body fat, and income variables for both groups of students, consumers at the University Restaurants.

**Variables**		**Group 1 (*n* = 79)**			**Group 2 (*n* = 94)**		
		n	%	*p*	*n*	%	*p*
Age Group	<20	21	26.6		24	25.5	
	20–29	54	68.4	0.000 **	63	67	0.001 **
	30–39	3	3.8		5	5.3	
	>40	1	1.3		2	2.1	
BMI (average ± SD)	kg/m²	21.8 ± 3.7		0.027 ***	23.2 ± 4.2		0.027 ***
Body fat percentage (mean)		20.7 ±8.9		0.411 ***	19.6 ±7.2		0.411 ***
Income	<1.5 mw *	79	100		-	-	
	>1.5 mw *	-	-	-	21	22.3	
	>3–5 mw *	-	-		10	10.6	0.001 **
	>5 mw *	-	-		28	29.8	
	Not Declared, but >1.5 mw	-	-		35	37.2	

* mw—Brazilian minimum wage (US$289.00); ** Kruskal–Wallis; ***chi-square.

Table S2. The Measure of central tendency and variance of the student's intake nutritional composition by frequency of consumption in the University Restaurant.

	**Consumption of 3 mains meals at University restaurant (Monday)**							**Consumption of 3 mains meals at University restaurant (Tuesday)**						
	YES			NO				YES			NO			
	Percentile 25	Median	Percentile 75	Percentile 25	Median	Percentile 75	P	Percentile 25	Median	Percentile 75	Percentile 25	Median	Percentile 75	P
**TEI (kcal/day)**	2629.8	3204.0	3859.0	1700.3	2460.6	3465.0	0.007*	2393.7	3208.3	3400.7	1749.0	2317.3	3400.6	0.022*
**Cholesterol (mg/day)**	115.8	203.1	270.0	129.8	227.9	364.3	0.442	186.3	206.3	232.1	93.6	194.7	303.5	0.917
**Fiber (g/day)**	56.6	80.5	120.7	35.8	57.0	79.2	0.025*	60.9	73.5	108.4	33.7	55.7	73.7	0.007*
**Iron (mg/day)**	18.3	20.5	37.8	13.6	18.1	25.6	0.023*	18.3	28.6	34.4	12.2	18.2	25.7	0.012*
**Sodium (mg/dia)**	4999.6	6591.9	7190.2	3287.3	4659.3	6485.5	0.009*	5795.7	6932.8	8689.8	3538.7	4768.7	6334.6	0.001*
**Calcium (mg/dia)**	848.4	1109.1	1475.9	469.6	665.6	1107.2	0.003*	788.5	1001.7	1225.7	502.5	732.4	956.3	0.013*

**Carbohydrate (% of TEI)**	59.9	64.9	70.8	55.2	59.	67.5	0.105	53.0	59.2	62.6	51.2	57.5	64.0	0.916
**Lipid (% of TEI)**	14.6	18.4	21.4	16.7	20.7	26.2	0.176	21.3	23.0	25.4	19.3	25.5	31.4	0.535
**Protein (% of TEI)**	13.9	17.7	18.8	14.4	17.6	20.1	0.675	16.2	17.7	19.3	14.7	16.9	19.7	0.472

* P<0.05; Mann-Whitney U test.
